# Processing Conditions, Thermal and Mechanical Responses of Stretchable Poly (Lactic Acid)/Poly (Butylene Succinate) Films

**DOI:** 10.3390/ma10070809

**Published:** 2017-07-16

**Authors:** Elena Fortunati, Debora Puglia, Antonio Iannoni, Andrea Terenzi, José Maria Kenny, Luigi Torre

**Affiliations:** Civil and Environmental Engineering Department, University of Perugia, UdR INSTM, Strada di Pentima 4, 05100 Terni, Italy; elena.fortunati@unipg.it (E.F.); antonio.iannoni@unipg.it (A.I.); andrea.terenzi@unipg.it (A.T.); jose.kenny@unipg.it (J.M.K.); luigi.torre@unipg.it (L.T.)

**Keywords:** poly (lactic acid), poly (butylene succinate), blend, plasticizers, stretchable films, processing, thermal properties, mechanical properties, wettability

## Abstract

Poly (lactic acid) (PLA) and poly (butylene succinate) (PBS) based films containing two different plasticizers [Acetyl Tributyl Citrate (ATBC) and isosorbide diester (ISE)] at three different contents (15 wt %, 20 wt % and 30 wt %) were produced by extrusion method. Thermal, morphological, mechanical and wettability behavior of produced materials was investigated as a function of plasticizer content. Filmature parameters were also adjusted and optimized for different formulations, in order to obtain similar thickness for different systems. Differential scanning calorimeter (DSC) results and evaluation of solubility parameter confirmed that similar miscibility was obtained for ATBC and ISE in PLA, while the two selected plasticizers resulted as not efficient for plasticization of PBS, to the limit that the PBS–30ATBC resulted as not processable. On the basis of these results, isosorbide-based plasticizer was considered a suitable agent for modification of a selected blend (PLA/PBS 80:20) and two mixing approaches were used to identify the role of ISE in the plasticization process: results from mechanical analysis confirmed that both produced PLA–PBS blends (PLA85–ISE15)–PBS20 and (PLA80–PBS20)–ISE15 could guarantee advantages in terms of deformability, with respect to the PLA80–PBS20 reference film, suggesting that the promising use of these stretchable PLA–PBS based films plasticized with isosorbide can provide novel solutions for food packaging applications.

## 1. Introduction

In recent years, environmental contamination has become a great concern due to the high impact of plastic waste in daily use. One of the possible solutions to this problem is to replace the commodity synthetic polymers with the biodegradable polymers, which are readily susceptible to microbial action. Their use is imposed by the need for replacement of conventional materials derived from petroleum sources, as well as from the viewpoint of environmental pollution prevention. Materials from biodegradable and non-toxic biocompatible polymers which are synthesized entirely or partially from annually renewable resources have drawn great interest [1]. Amongst all, the biocompatible and thermoplastic poly (lactide) PLA is one of the most promising biobased polymers [2], with applications ranging from drug-carriers and implants, to packaging and textiles.

Poly (lactic acid) (PLA), a linear aliphatic polyester produced from renewable resources, has attracted much attention in recent years, due to its biodegradability, and could well be a possible solution for solid waste [3]. PLA has good mechanical, thermal and biodegradable properties and is, therefore, a good polymer for various end-use applications [4]. However, other properties like flexural properties, heat distortion temperature (HDT), gas permeability, impact strength, melting viscosity for processing, etc. are not good enough for applications such as packaging [5]. Additionally, high price and brittleness of PLA lowers the possibility of its commercialization. Therefore, blending PLA with other suitable biodegradable polymers, which have comparably better flexural properties, excellent impact strength and melt processability, will modify various properties and also contribute towards low overall material cost. 

Poly (butylene succinate) (PBS) is a biodegradable aliphatic polyester produced by the polycondensation reaction of 1,4-butanediol with succinic acid [6,7] with high flexibility, excellent impact strength, and thermal and chemical resistance. It can be processed easily and represents the best choice to blend with PLA. Previous work on the properties of PLA blends with biodegradable polymers has reported that in PLA/PBS blends both polymers were incompatible at more than 20 wt % of PBS, resulting in a decreased tensile strength, modulus and percentage (%) elongation at break, with increasing PBS content [8]. Lui et al. [9] blended PLA with poly (ethylene/butylene succinate) (Bionolle, Munich, Germany) and found that adding Bionolle aided in crystallization of PLA. Bionolle concentration led to a slight increase in the strain-at-break of the blends, but a decrease in the Young's modulus and ultimate tensile strength. Yokohara and Yamaguchi [10] studied the structure and properties of binary blends composed of poly (lactic acid) (PLA) and fibrous poly (butylene succinate) (PBS) and compared with those of blends having spherical particles of PBS in a continuous PLA phase. PBS particles, which showed nucleating ability for PLA, led to a high degree of crystallinity and enhanced the cold crystallization in the heat process. Sung et al. [11] reported that the use of PBS increases the crystallization rate of PBS/PLA blends. Zhou et al. [12] reported that the binary systems of PBS/PLA are finely dispersed immiscible blends exhibiting phase separation. In fact, the introduction of branching structures in PLA is an effectual method to overcome the disadvantages of PLA [13].

The addition of plasticizers is another way to overcome the brittle nature of PLA and improve its flexibility [14]. An idealistic plasticizer for PLA is expected to be miscible with the polymer matrix, biodegradable, non-volatile and nontoxic. Up to now, a large number of low-molecular-weight compounds have already been used as potential plasticizers for PLA: although these plasticizers could reduce the brittleness of PLA and may broaden its potential applications, most of them exhibit poor compatibility with PLA and phase separation was observed with the plasticizer concentration of 20 wt %, which limits their potential application in food packaging [15]. Furthermore, most of these plasticizers are relied on unsustainable crude oil, and the extensive use of them would bring great pressure to the increasing shortage of petroleum resources and the environmental problems. Therefore, it is of great significance to exploit renewable resources for developing “green” plasticizers. Isosorbide esters are a class of plasticizers entirely based on renewable resources, which are synthesized from isosorbide, a dehydration product of glucose-derived sorbitol. Their properties can be facilely tuned by using different alkanoic acids. More importantly, isosorbide esters are fully biodegradable and have passed tests for acute toxicity, sensitization, mutagenicity, and estrogenicity [16].

Recent works on isosorbide containing polymers have included the development of starch [17,18], PVC [19,20,21] and PLA based systems [22,23,24].

However, to the best knowledge of the authors, no work has been done regarding the use of isosorbide as plasticizer for polybutylene succinate and/or its blend with PLA. Thus, this paper was focused on the possibility of obtaining isosorbide-based PLA–PBS blends and on the investigation of properties for possible use in the packaging field. Special attention was given to the processing overview with respect to a common plasticizer: for this reason, PLA–PBS systems with acetyl tributyl citrate as plasticizer (ATBC) were prepared as a reference.

## 2. Materials and Methods

### 2.1. Materials

Poly (lactic acid) (PLA) 3051D, with a specific gravity of 1.25 g·cm^−3^, a molecular weight (*M*_n_) of ca. 1.42 × 10^4^ g·mol^−1^, and a melt flow index (MFI) of 7.75 g 10 min^−1^ (210 °C, 2.16 kg) was supplied by NatureWorks^®^, Minnetonka, MN, USA. Poly (butylene succinate) (PBS—under the trade name PBE 003) with a specific gravity of 1.26 g·cm^−3^, a molecular weight (*M*_n_) of ca. 1.42 × 10^4^ g·mol^−1^, and a melt flow index (MFI) of 4.5 g 10 min^−1^ (210 °C, 2.16 kg) was supplied by NaturePlast^®^, Ifs, France. Acetyl tributyl citrate (ATBC, *M* = 402 g·mol^−1^, 98% purity) was purchased from Sigma-Aldrich^®^, Milan, Italy whereas Polysorb (ISE—under the trade name ID46-Diester 93.2%) was supplied by Roquette^®^, (Lestrem, France).

### 2.2. Preparation of PLA and/or PBS Based Plasticized Films

#### 2.2.1. Optimization of Plasticizer Contents in PLA or PBS Based Films

**Figure 1:**
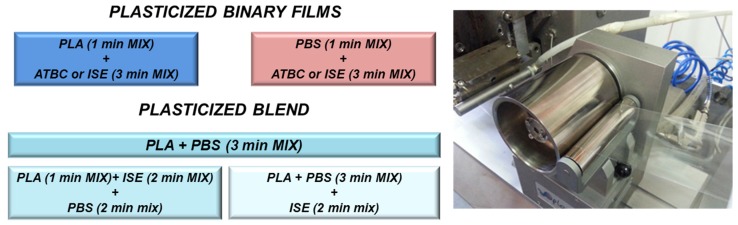
Film forming procedure.

PLA or PBS based plasticized formulations, produced by adding ATBC or ISE at different contents, were manufactured by using a twin-screw microextruder (DSM explorer 5&15 CC Micro Compounder, Xplore Instruments BV, Sittard, The Netherlands) at screw speed of 100 rpm and mixing time of 4 min. PLA pellets were dried overnight at 50 °C, whereas PBS was dried in vacuum for 12 h at 70 °C. The plasticizers, ATBC and ISE, were dried overnight at 80 °C before processing. PLA and PBS based master batches were previously prepared by adding ATBC or ISE as plasticizers for the two selected polymeric matrices. Specifically, PLA or PBS pellets were put in the microextruder to manually reach a head force of around 1000–1200 N, by using a mixing temperature profile characterized by means of a three-step temperature procedure of 180–190–200 °C for PLA matrix, and 130–135–140 °C for PBS polymer, as well as a screw speed of 100 rpm for all the produced systems. After 1 min of mixing of PLA or PBS, ATBC or ISE were added into the microextruder for 3 additional minutes. Three different weight ratios adding 15 wt %, 20 wt % or 30 wt % of ATBC or ISE into PLA or PBS matrix were considered. Directly after the mixing process, a film forming process was conducted. Preformed PLA–ATBC or PLA–ISE and PBS–ATBC or PBS–ISE, with plasticizers at different amounts, granulated into pellets, were melted again in the microextruder and the film processing was applied according to the parameters reported in Table 1 at 30 rpm for all formulations. Plasticized films, with a thickness ranged from 20 to 50 µm, were obtained and designed as shown in Table 1. Neat PLA and PBS films were also produced for comparison. Some filmature parameters, especially Speed, Torque and Head force were adjusted and optimized for different formulations (see Table 1) in order to obtain similar thickness for different systems. Figure 1 shows an example of film forming procedure.

#### 2.2.2. Processing and Optimization of Plasticized PLA–PBS Based Blends

PLA–PBS plasticized blend films were produced with 80 wt % of PLA and 20 wt % of PBS on the basis of previous work [25]. The type and content of plasticizer were selected on the base of mechanical and thermal data obtained for PLA or PBS based plasticized films. Specifically, ISE at 15 wt % of the total amount of the polymers was here used as plasticizer for PLA–PBS based films. Masterbatches were firstly prepared also in this case by using the twin-screw microextruder (DSM explorer 5&15 CC Micro Compounder) and applying the same step temperature of 180–190–200 °C and the screw speed of 100 rpm, as previously noted for PLA or PBS based plasticized films. However, two mixing approaches were performed in order to optimize the processing: in the first case, after 1 min of mixing of PLA, ISE was added into the microextruder for 2 min in order to permit the interaction of the plasticizer with PLA matrix firstly, and finally PBS was added and mixed for 2 additional minutes; in the second approach, PLA and PBS for firstly mixed for 3 min (80 wt % of PLA and 20 wt % of PBS, respectively) and then 15 wt % of ISE was added for the last 2 mixing minutes. Also in this case, after the mixing process, the obtained master batches were granulated into pellets, and a film processing was applied according to the parameters reported in Table 2 at 30 rpm. Plasticized PLA–PBS based films with a thickness ranged from 20 to 50 µm and produced by the above approaches, were obtained and designed as in Table 2, whereas neat PLA80–PBS20 blend film were produced for comparison. Also in this case, as previously said, Speed and Torque were adjusted and optimized during the filmature step (see Table 2) in order to obtain similar thickness for different systems.

### 2.3. Characterization Techniques

Different characterization methods were applied in order to select firstly the appropriate content and the best type of plasticizer, taking into account the final possible application of the produced formulations as stretchable films for industrial application. Specifically, thermal and mechanical analyses were conducted for PLA or PBS plasticized formulations and then also performed for to the PLA–PBS based blend films produced with the optimized content and type of plasticizer.

Thermal behavior of produced films was tested by means of a differential scanning calorimeter (DSC, TA Instrument, Q200, New Castle, DE, USA). Measurements were carried out under nitrogen flow in the temperature range from −25 to 220 °C at a heating rate of 10 °C/min. After a first heating step, cooling and second heating were performed. Data were recorded both during the cooling and second heating steps. The glass transition temperature (*T*_g_) was taken as the inflection point of the specific heat increment at the glass–rubber transition, while the melting temperature (*T*_m_) and melting enthalpy (Δ*H*_m_) were determined during the cooling and the 2nd heating scan, respectively. Three samples were used to characterize each material. The crystallinity degree (*X*_c_) was calculated as reported in Equation (1):

(1) $$X_{c}=\frac{\Delta H_{m}}{\Delta H_{0}\left(1-m_{f}\right)}\times 100$$

where Δ*H*_0_ is enthalpy of melting for a 100% crystalline sample, taken as 94 J/g [26] and 110.3 J/g [27], respectively in the case of PLA and PBS, and (1 − m_f_) is the weight fraction of polymer in the sample.

The mechanical behavior of different produced formulations was investigated by tensile test in a digital Lloyd instrument LR 30 K (Bognor Regis, UK), performed on rectangular probes (200 mm × 25 mm) by following the EN ISO 527: 2012 standard [28] with a crosshead speed of 10 mm/min, a load cell of 500 N and an initial gauge length of 100 mm. Average tensile strength (σ_B_) and percentage elongation at break (ε_B_), yield stress and strain (σ_Y_, ε_Y_) and elastic modulus (*E*) were calculated from the resulting stress–strain curves. The measurements were done at room temperature and at least five samples for each material were tested.

The microstructure of film cross-sections was observed by using a Field Emission Scanning Electron Microscope, FESEM, Supra 25-Zeiss, Oberkochen, Germany. Specimens were cryo-fractured by immersion in liquid nitrogen and mounted on copper stubs perpendicularly to their surface. Samples were gold coated and observed by using an accelerating voltage of 4 kV.

Finally, surface wettability of the PLA–PBS based blend films produced with the optimized content and type of plasticizer, was studied through static water contact angle measurements with a standard goniometer (FTA2000, First Ten Angstroms, Inc. Portsmouth, UK) equipped with a camera and Drop Shape Analysis SW21; FTA32 2.0 software (First Ten Angstroms, Inc., Portsmouth, UK) was used to test the water contact angle (θ°) at room temperature. The contact angle was determined by randomly putting 5 drops of distilled water (≈2 μL) with a syringe onto the film surfaces and, after 30 s, the average values of ten measurements for each drop were used.

## 3. Results and Discussion

### 3.1. Thermal Properties

**Figure 2:**
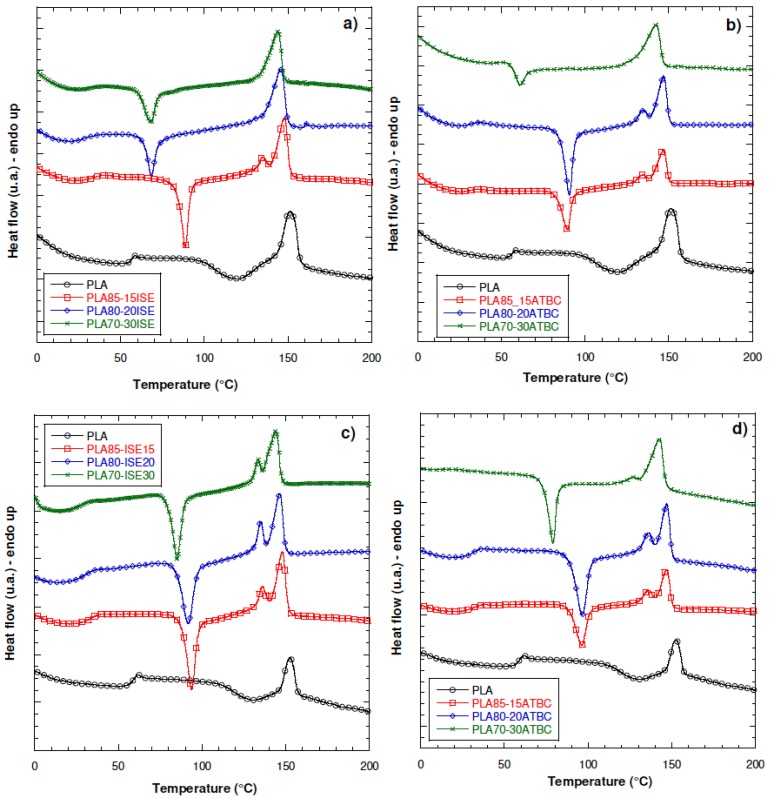
Differential scanning calorimeter (DSC) thermograms of PLA modified with isosorbide diester (ISE) ((**a**,**c**), first and second heating scan) and Acetyl Tributyl Citrate (ATBC) ((**b**,**d**), first and second heating scan) at the three different weight content of 15 wt %, 20 wt % and 30 wt %.

The DSC thermograms related to the first heating scan of PLA modified with the two different plasticizers are presented in Figure 2a,b, respectively for ISE and ATBC. The corresponding thermal data are listed in Table 3.

The addition of ISE plasticizer caused marked changes in the crystallization behavior of the PLA matrix. Moreover, as the amount of plasticizer increased, the glass transition temperature (*T*_g_), cold crystallization, melting point and melting enthalpy of PLA shifted to lower temperatures. The decrease in *T*_g_, cold crystallization and melting peaks were due to segmental mobility of PLA chains caused by the presence of the plasticizer, increasing with the plasticizer content which indicates higher polymer flexibility [23,29]. Such results proved the good plasticizing effect induced by ISE.

The glass transition difference between PLA80–ISE20 and PLA70–ISE30 blends was limited, and it was attributed to the saturation of ISE solubility at 20 wt % in PLA, that caused the considerable limitation of *T*_g_ decrement. Besides the glass transition, traces of cold crystallization and subsequent melting peaks are also detected on the thermograms of all samples. It is clear that the cold crystallization temperatures (*T*_cc_) of the PLA/ISE samples shifted to lower temperatures, while two melting peaks were found in the DSC thermograms of PLA/ISE blends with ISE concentration at 15 wt %, and it can be attributed to the formation of crystallites with different lamellar thicknesses and crystal perfection, which is arisen by the lamellar rearrangement [30,31]. As the content of ISE reaches 20 wt %, the lower melting peak disappeared, which means that the mobility of PLA chains is quite high. In the case of PLA/ATBC, based formulations the saturation effect of the plasticizer was reached at higher content (20 wt %). By increasing the content of the plasticizer, the effectiveness of the ATBC plasticizer to reduce the *T*_c_ of the PLA, with respect to ISE, was generally enhanced with respect to ISE. The addition of ISE or ATBC to the PLA affected also the cold crystallization temperature (*T*_cc_). The *T*_cc_ observed in treated PLA at 120.7 °C was depressed to 66.9 °C and to 61.6 °C with addition of 30 wt % of ISE and ATBC, respectively. The increase of the degree of crystallinity, as shown in Table 3, strongly depends on plasticizer content. The percentage of crystallinity of plasticized PLA was, in general, higher with respect to the unmodified PLA. For the PLA/ISE plasticized films, greatest crystallinity was observed with an addition of 30 wt % for a value of 27.3%, and a value of 21.9% for the plasticized PLA/ATBC of the same content. This increase indicates that the crystallization of the PLA becomes easier with the mobility of the chains caused by the isosorbide ester, essentially due to the low molecular weight of ISE (174 g·mol^−1^) in comparison to ATBC (402 g·mol^−1^).

In the second heating scan (Figure 2c,d), the thermal behavior of the analyzed systems was substantially maintained, suggesting that the presence of ATBC facilitated crystal nucleation process, which resulted in a lower value for cold crystallization temperature (78.9 °C and 84.4 °C for ATBC and ISE at 30%, respectively). At the higher plasticizer concentration, the films plasticized by ATBC showed a lower glass transition temperature as compared to those containing ISE, probably due to the more flexible structure of the plasticizer which generally leads to lower glass transition temperature. Two melting peaks have been found also in the second heating curves for all the PLA/ISE and PLA/ATBC systems, confirming the presence of two populations of lamellae, the small ones produced by secondary crystallization and major crystals formed in the primary crystallization process [32].

The reason for good solubility of the two plasticizers in PLA is due to the polar interactions between the ester groups of PLA and the plasticizers themselves [33]. It is believed that the most effective plasticizers for a specific polymer could be those with a similar structure to the polymer matrix and the miscibility can be evaluated by comparing their solubility parameters [15]. In this study, the solubility parameters for PLA, PBS, ISE and ATBC were calculated according to the Small’s cohesive energies by Equation (2) [34]:

(2) $$\delta =\frac{\rho \sum F_{1}}{M_{o}}$$

where *δ* is the solubility parameter, *ρ* is the density, *M_o_* is the molecular mass and Σ*F*_1_ is the sum of the group molar attraction constants estimating the contribution of each group in their chemical structure. The calculated results are listed in Table 4, wherein we can see that ATBC had a solubility parameter closer to PLA than that of ISE. Therefore, it was reasonable to speculate that ATBC would have better miscibility with PLA than ISE.

Looking at the results of solubility parameter calculation, it is also evident how the two selected plasticizers could result theoretically not efficient for plasticization of polybutylene succinate, while it is confirmed how PBS represents a good selection for attempt to modify brittle poly (lactic acid) into a flexible matrix [35]. Only a few examples can be found in the literature for PBS direct plasticization, where the use of epoxidized soybean oil (ESO) in the range 2.5–17.5 wt % modified the thermal behavior of the neat matrix, by lowering glass transition, crystallization and melting temperatures [36]. Even with ESO, a limited compatibility with PBS was revealed and phase separation was observed when more plasticizer was added.

**Figure 3:**
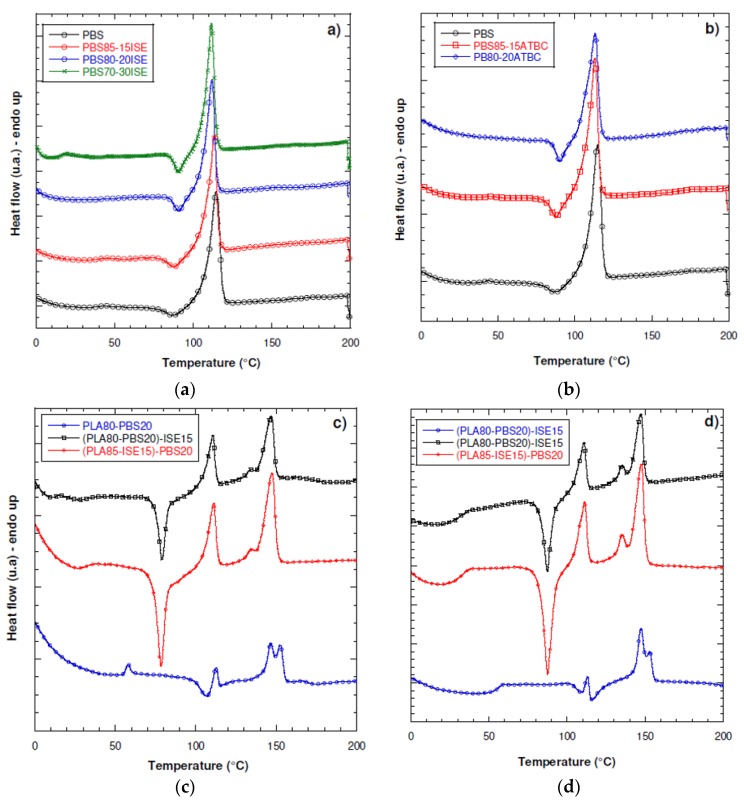
DSC thermograms (first heating scan) of PBS modified with (**a**) ISE and (**b**) ATBC at the three different weight contents of 15 wt %, 20 wt % and 30 wt %; (**c**) first heating scan and (**d**) second heating scan of PLA80–20PBS blend and ternary systems modified with ISE at 15 wt % [(PLA85–ISE15)–PBS20 and (PLA80–PBS20)–ISE15)].

In our case, results from Figure 3a,b and Table 5 confirm the substantial weak effect of ISE and ATBC when introduced in PBS in all the different weight content, to the limit that the PBS–30ATBC resulted not processable and, consequently, it was not possible to fully characterize this system. On the basis of these results, isosorbide plasticizer was considered as a suitable agent for modification of PLA80–PBS20 blend. Figure 3c,d shows the first and second heating DSC scans for the PLA80–PBS20 blend and their system plasticized with isosorbide, by using the approach described in Section 2.2.2, (PLA85–ISE15)–PBS20 and PLA80–PBS20)–ISE15. While PLA films show only one melting peak, as already observed in Figure 2a, PLA80–PBS20 based films show two melting peaks, indicating that each polymeric component crystallizes individually and the polymeric blends are immiscible [8]. While neat PLA displayed a *T*_g_ at 58.0 °C, a *T*_cc_ at 120.7 °C and a *T*_m_ at 150.9 °C, *T*_cc_ and *T*_m_ were observed, for the pure PBS, respectively, at 87.4 and 114.0 °C (Table 5). Thus, PBS can crystallize much more easily than PLA. In the PLA80/PBS20 blend, the *T*_g_ of the PLA component (55.3 °C) was close to that of pure PLA, indicating that PLA and PBS were thermodynamically incompatible [37]. In addition, only a slight increase in the *T*_m_ of PLA (153.0 °C) was observed, so the presence of PBS did not significantly influence the *T*_m_. A decrease in the *T*_cc_ of PLA in the blend was noted (106.6 °C) compared to that of pure PLA, which could be due to the increased mobility of the PLA chains after blending with PBS, due to the strong plasticization and nucleating effects of PBS in accelerating PLA chain alignment [10]. For PLA/PBS blends containing ISE at 15 wt %, a similar behaviour was found for the two studied system, with no particular deviation for the two adopted master batch procedures. Melting temperatures of PLA in the two systems (PLA85–ISE15)–PBS20 and (PLA80–PBS20)–ISE15 were slightly lower than those in the PLA80–PBS20 blend (134.6 °C and 134.5 °C for *T*_m1_, 147.3 °C and 146.8 °C for *T*_m2_ for (PLA85–ISE15)–PBS20 and (PLA80–PBS20)–ISE15), respectively, in comparison with PLA80–PBS20, having 147 °C and 153 °C. It can be deduced that the mobility of the polyester segments in these blends may have been enhanced by the ISE presence. Moreover, the addition of the isosorbide into the PLA/PBS blend had a valuable effect on PLA glass transition, moving from 55.3 °C up to 34.3 °C and 32.8 °C for (PLA85–ISE15)–PBS20 and (PLA80–PBS20)–ISE15. As expected, the *T*_cc_ of PLA in all the plasticized systems was further shifted towards lower temperatures than that in the 80/20 PLA80–PBS20 blend (similarly at 79.0 °C both for (PLA85–ISE15)–PBS20 and (PLA80–PBS20)–ISE15).

A similar behavior was recorded in the second heating scan (Figure 3d), with only an inversion in the amplitude of melting peaks for PLA/PBS blend at T_m1_ and T_m2_, speculating an inversion in preponderant existence of thinner lamellae with respect of thicker ones, while no effect has been registered for melting profile of the two plasticized systems (PLA85–ISE15)–PBS20 and (PLA80–PBS20)–ISE15 with respect of first heating scan.

### 3.2. Mechanical Behaviour

**Figure 4:**
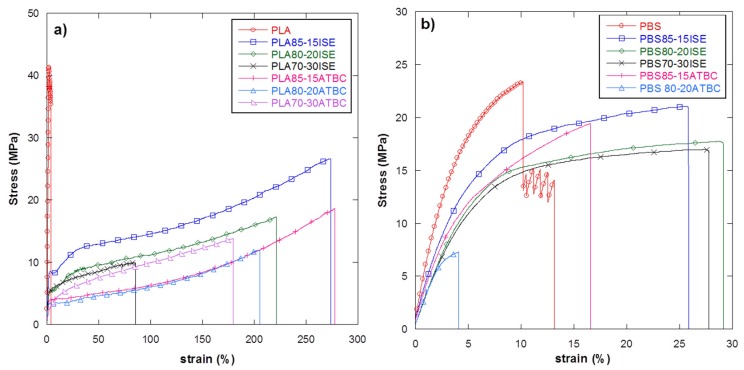
Stress–strain curves for different (**a**) PLA and (**b**) PBS films plasticized with ISE and ATBC at the three different weight contents of 15 wt %, 20 wt % and 30 wt %.

Mechanical tests were firstly conducted on PLA or PBS based films, plasticized with both ATBC and ISE at three different contents (15 wt %, 20 wt % or 30 wt %) in order to select the best amount of plasticizer and compare the effect of a greener and novel molecule, ISE, with respect to the just known and used ATBC [38,39], on the elongation properties of both PLA and PBS polymeric matrices. The results from tensile tests are summarized in Table 6, whereas Figure 4a,b shows the typical stress–strain curves for different PLA or PBS plasticized films.

**Figure 5:**
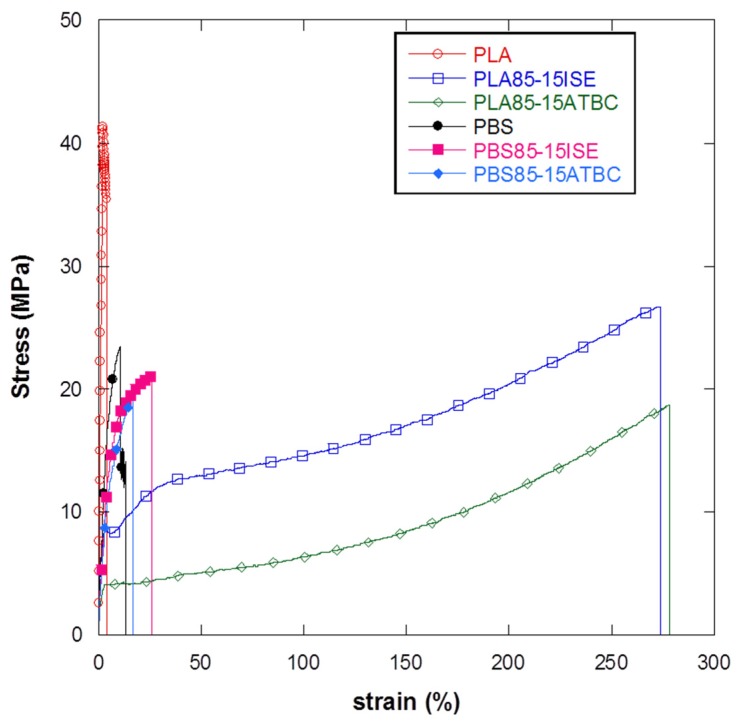
Comparison of curves from tensile tests PLA and PBS films plasticized with ISE and ATBC at 15 wt %.

The incorporation of ATBC causes, as expected, a reduction in Young’s modulus and an evident increase of the elongation at break of the PLA films that is particular evident at 15 wt % and 20 wt % of plasticizer reaching (228.6 ± 59.9)% and (199.1 ± 5.6)% respect. A similar behaviour was also registered for the ISE plasticizer with (208.6 ± 58.4)% and (196.1 ± 35.3)% of elongation for PLA85–ISE15 and PLA80–ISE20, respectively. These results, in accord with the previous thermal analysis, display a significant improvement in the mobility of the chains with a consequent increase in the flexibility of PLA due of the plasticization effect achieved by plasticizer addition. In PLA matrix, a comparable effect of both plasticizers on elongation at break properties was registered; however, ISE allows for maintaining a better overall mechanical response since higher elastic modulus and tensile strength were guaranteed by its addition, with respect to ATBC based films (Table 6 and Figure 4a). An interesting effect of ISE was also registered when added, at different contents, in the PBS matrix. The incorporation of ISE in this polymeric matrix causes, as previously for PLA, a reduction in Young’s modulus and an increase of the elongation at break (Table 6 and Figure 4b), although lower deformation values were registered for PBS based plasticized films respect to PLA based systems. A similar behaviour has been found by other authors also with other plasticizers [36]. Moreover, less effect of ATBC as plasticizer for PBS was highlighted by mechanical tests, with a modest increase in the elongation at break value (14.6 ± 1.2)% for PBS85–ATBC15 films, with respect to a neat PBS based system (9.7 ± 1.9)% and a reduction in the deformation value registered for PBS85–ATBC20, with respect to the 15 wt % based ATBC formulation. Finally, it is interesting to note that for both plasticizers added to PLA or PBS matrices, 15 wt % represent a sort of saturation limit since higher contents do not correspond to an increase in elongation at break values (Figure 5).

**Figure 6:**
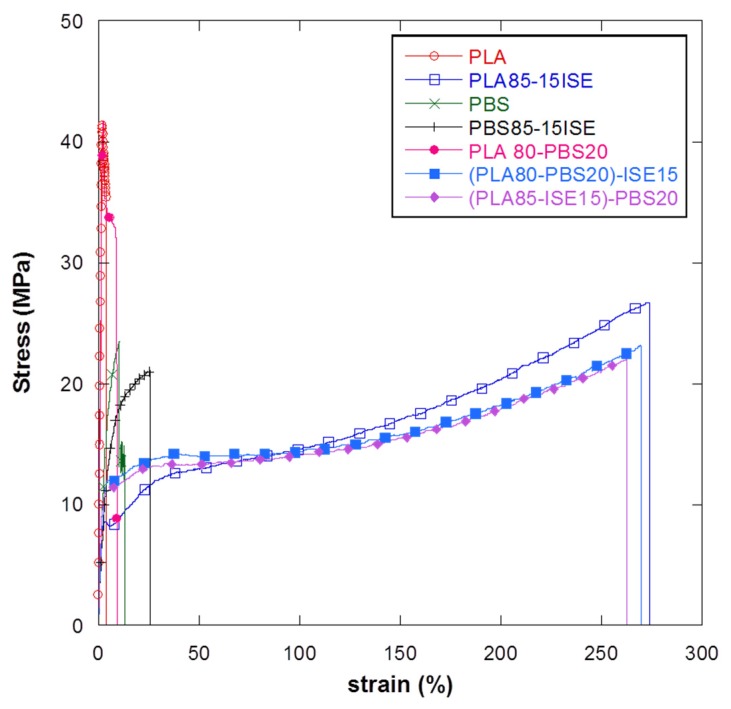
Stress–strain curves for films obtained from PLA80–20PBS and PLA–PBS blends plasticized with ISE at 15 wt %by two different mixing procedures ((PLA85–ISE15)–PBS20 and (PLA80–PBS20)–ISE15).

On the basis of these results, also combined with thermal data, the ISE plasticizer and its content fixed at 15 wt % with respect to the total amount of the polymers, appear as the best choices for the next production of PLA–PBS plasticized blend films and (PLA15–ISE15)–PBS20 and (PLA80–PBS20)–ISE15, produced by the two different mixing approaches, were tested and compared with PLA80–PBS20 reference formulation. The results from tensile test are summarized in Table 6 whereas the obtained stress–strain curves for blends are shown in Figure 6.

Data underlined that a comparable behavior was found for PLA–PBS blends respect to PLA plasticized films in terms of mechanical responses. The presence of ISE at 15 wt %, in fact, guaranteed an evident increase in the elongation at break values (241.6 ± 37.6)% for (PLA15–ISE15)–PBS20 and (249.5 ± 19.4)% for (PLA80–PBS20)–ISE15 with respect to the (10.5 ± 5.4)% of the PLA80–PBS20 reference blend film, with a consequent decrease in the elastic modulus values as expected. Furthermore, no particular differences were detected in terms of mechanical behaviour between (PLA85–ISE15)–PBS20 and (PLA80–PBS20)–ISE15 systems produced by the two different mixing approaches. In conclusion, the results from the mechanical analysis confirmed that both the produced PLA–PBS blends, plasticized with the novel greener route, could guarantee advantages in term of the deformability, as previously proved for PLA based plasticized systems. However, at the same time, the presence of PBS could maintain a high level of gas barrier just proved in similar PLA–PBS base systems [27], which is useful for the final practical application at an industrial level (i.e., packaging sector).

### 3.3. Microstructure of Produced Films

**Figure 7:**
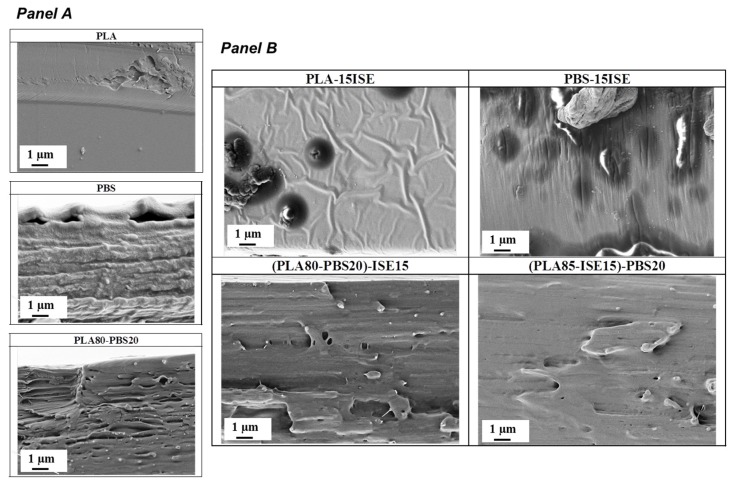
FESEM images of PLA, PBS and PLA80–PBS20 fractured surfaces (**Panel A**) and of PLA, PBS or PLA–PBS blends plasticized with ISE at 15 wt % by two different mixing procedures ((PLA85–ISE15)–PBS20 and (PLA80–PBS20)–ISE15) (**Panel B**).

The fractured surfaces (cross-sections) of PLA, PBS and PLA80–PBS20 blend were firstly investigated by FESEM (Figure 7, Panel A) to evaluate the effect of PBS presence and content in PLA matrix microstructure. A characteristic smooth fractured surface was observed for PLA neat film, whereas a rougher surface characterized the cross section of PBS based film. PLA80–PBS20 formulation exhibited the typical morphology of this polymeric combination, with a phase separation and with the typical formation of nodules and a sort of porous structure induced by the presence of PBS at this content in PLA matrix, as previously observed in the literature [27].

The fractured surfaces of PLA or PBS plasticized with the selected best content (15 wt %) of ISE and the produced ternary plasticized blends were also investigated and the results reported in Figure 7B. The plasticization effect exerted by ISE in both PLA and PBS matrix was clearly evidence by FESEM images, confirmed the previous discussed thermal and mechanical data.

Furthermore, the positive effect of ISE presence was strongly evident in both (PLA85–ISE15)–PBS20 and (PLA80–PBS20)–ISE15 based films, that showed a more homogeneous morphology respect to the PLA80–PBS20 reference blend film, with a reduction of the porous structure and less of a presence of holes and defects. FESEM images confirmed the plasticization effect of ISE in both produced PLA80–PBS20 based blends, underlining the positive effect of this plasticizer in the compatibility increase between the two selected polymeric matrices, as discussed in thermal property section.

### 3.4. Contact Angle Studies of PLA–PBS Based Films

Surface wettability of PLA, PLA85–ISE15 and of PLA–PBS based blend films produced with the optimized content and type of plasticizer, was studied in order to evaluate the effect of both PBS and plasticizers addition on the surface properties of PLA based film. Wettability properties are, in fact of interest for specific industrial sector as i.e., packaging fields, where the printability of the surfaces, directly related to the surface wettability, is strongly required by both producers and consumers. The hydrophilicity of the samples was investigated by water contact angle measurement and the results summarized in Table 7. When ISE was added to the PLA matrix at 15 wt %, the contact angle with water does not change, whereas PBS addition provoked a decrease from about 68° for neat PLA to 61° for PLA80–PBS20. Moreover, an either decrease was registered when 15 wt % of ISE was added to the PLA–PBS blend, reaching 56° of CA values for both (PLA85–ISE15)–PBS20 and (PLA80–PBS20)–ISE15 formulations.

## 4. Conclusions

Different characterization methods were applied in order to select firstly the appropriate content and the best type of plasticizer, between ATBC and ISE, taking into account the final possible application of the produced formulations as stretchable films for the packaging sector. Specifically, thermal and mechanical analyses were conducted for PLA or PBS plasticized formulations. Thermal studies by differential scanning calorimeter (DSC) underlined that both ATBC and ISE were able to decrease both the glass transition temperature and the cold crystallization temperature for PLA plasticized films, whereas a reduced effect was noticed for PBS plasticized films. Moreover, the best performances were found for ISE used at 15 wt % and similar results were also obtained by mechanical tests: ISE used at 15 wt % provoked a reduction in Young’s modulus and an increase of the elongation at break both in PLA or PBS based films suggesting that ISE at 15 wt % represents the best choice for the production of PLA–PBS plasticized blend films. (PLA85–ISE15)–PBS20 and (PLA80–PBS20)–ISE15 systems confirm the plasticization effect of ISE at 15 wt %, that is able to guarantee an evident increase in the elongation at break values, with respect to the PLA80–PBS20 reference film. This plasticization effect was also confirmed by an increase in surface wettability registered for PLA80–PBS20 plasticized blends. The obtained results suggested the promising use of these stretchable PLA–PBS based films plasticized as a novel green route solution for food packaging applications.

## Figures and Tables

**Table 1:** Poly (lactic acid) (PLA) or poly (butylene succinate) (PBS) based plasticized formulations: materials designation and filmature conditions.

Formulations	Temperature Profile (°C)	Head Temperature (°C)	Filmature Parameters	Head Force (N)
PLA based plasticized films				
PLA	180–190–200	195	Speed = 300 rpm; Torque = 50 N·m	800
PLA85–ATBC15	180–190–200	185	Speed = 450 rpm; Torque = 50 N·m	500
PLA80–ATBC20	180–190–200	180	Speed = 470 rpm; Torque = 50 N·m	600
PLA70–ATBC30	180–190–200	170	Speed = 470 rpm; Torque = 50 N·m	600
PLA85–ISE15	180–190–200	185	Speed = 450 rpm; Torque = 50 N·m	500
PLA80–ISE20	180–190–200	185	Speed = 450 rpm; Torque = 50 N·m	600
PLA70–ISE30	180–190–200	170	Speed = 450 rpm; Torque = 50 N m	400
PBS based plasticized films				
PBS	130–135–140	135	Speed = 450 rpm; Torque = 50 N·m	500
PBS85–ATBC15	130–135–140	130	Speed = 500 rpm; Torque = 50 N·m	500
PBS80–ATBC20	130–135–140	125	Speed = 600 rpm; Torque = 50 N·m	500
PBS70–ATBC30	130–135–140	125	Speed = 600 rpm; Torque = 50 N·m	500
PBS85–ISE15	130–135–140	135	Speed = 450 rpm; Torque = 50 N·m	500
PBS80–ISE20	130–135–140	120	Speed = 600 rpm; Torque = 50 N·m	500
PBS70–ISE30	130–135–140	120	Speed = 730 rpm; Torque = 50 N·m	500

**Table 2:** PLA–PBS based plasticized formulations: material designation and filmature conditions.

Formulations	Temperature Profile (°C)	Head Temperature (°C)	Filmature Parameters	Head Force (N)
PLA–PBS plasticized blend films				
PLA80–PBS20	180–190–200	205	Speed = 622 rpm; Torque = 50 N·m	500
(PLA85–ISE15)–PBS20	180–190–200	205	Speed = 787; Torque = 50 N·m	500
(PLA80–PBS20)–ISE15	180–190–200	205	Speed = 787 rpm; Torque = 50 N·m	500

**Table 3:** Thermal properties of PLA and PLA formulations plasticized with ISE and ATBC at different contents.

Formulations	*T*_g_ (°C)	*T*_cc_ (°C)	Δ*H*_cc_ (J/g)	*T*_m1_ (°C)	*T*_m2_ (°C)	Δ*H*_m_ (J/g)	*X*_c_ (%)	*T*_g_ (°C)
1st Heating Scan								Cooling
PLA	58.1 ± 0.4	120.7 ± 1.5	19.7 ± 2.2	-	150.9 ± 0.2	35.4 ± 0.5	16.7 ± 2.8	52.8 ± 2.0
PLA85–ATBC15	30.8 ± 0.7	89.4 ± 0.1	30.0 ± 0.1	134.2 ± 0.2	146.5 ± 0.1	32.9 ± 0.1	3.1 ± 0.2	25.5 ± 2.3
PLA80–ATBC20	31.8 ± 0.2	90.1 ± 0.6	28.5 ± 1.2	134.5 ± 0.3	146.4 ± 0.1	30.1 ± 0.1	2.1 ± 1.7	24.1 ± 0.5
PLA70–ATBC30	13.4 ± 3.0	61.6 ± 1.0	12.8 ± 1.0	-	142.3 ± 0.2	27.3 ± 0.1	21.9 ± 1.6	6.0 ± 0.7
PLA85–ISE15	34.8 ± 5.0	85.9 ± 3.8	25.6 ± 1.8	135.0 ± 0.5	147.7 ± 0.1	35.6 ± 0.6	10.7 ± 3.0	23.8 ± 1.5
PLA80–ISE20	29.4 ± 1.2	67.5 ± 0.6	22.8 ± 3.4	-	145.3 ± 0.2	38.6 ± 3.0	21.0 ± 0.5	23.4 ± 0.7
PLA70–ISE30	28.6 ± 2.7	66.9 ± 1.5	21.1 ± 0.3	-	143.0 ± 0.8	39.1 ± 0.7	27.3 ± 1.5	21.0 ± 1.1
2nd Heating Scan								
PLA	61.5 ± 0.2	129.6 ± 0.7	12.8 ± 1.6	-	152.0 ± 0.7	15.1 ± 3.0	2.5 ± 1.5	
PLA85–ATBC15	31.7 ± 0.6	96.1 ± 0.1	27.2 ± 3.0	135.7 ± 0.1	147.0 ± 0.4	32.5 ± 1.6	5.7 ± 1.7	
PLA80–ATBC20	30.5 ± 0.9	95.8 ± 0.3	24.7 ± 4.4	135.9 ± 0.6	146.7 ± 0.6	27.4 ± 3.9	3.6 ± 0.7	
PLA70–ATBC30	14.3 ± 2.5	78.9 ± 0.1	20.5 ± 1.2	127.0 ± 0.2	142.6 ± 0.1	26.5 ± 0.5	9.0 ± 2.6	
PLA85–ISE15	32.5 ± 0.1	93.7 ± 0.3	27.1 ± 0.8	136.1 ± 0.1	147.7 ± 0.1	33.0 ± 1.2	6.2 ± 2.5	
PLA80–ISE20	30.2 ± 0.5	91.6 ± 0.1	26.9 ± 1.1	134.8 ± 0.2	146.1 ± 0.1	30.5 ± 1.1	4.8 ± 3.0	
PLA70–ISE30	26.5 ± 0.6	84.4 ± 0.2	21.0 ± 0.9	133.5 ± 0.3	143.9 ± 0.2	27.8 ± 0.8	10.5 ± 0.1	

**Table 4:** Solubility parameters (δ) for all materials calculated with group molar attraction constants.

Materials	Chemical Formula	ρ (g/cm^3^)	δ (J/cm^3^)^1/2^
ATBC-Acetyl Tributyl Citrate	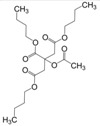	1.046	19.2
ISE-Isosorbide dimethyl ether	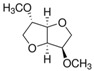	1.15	14.7
PLA	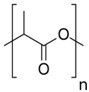	1.24	19.4
PBS	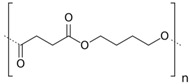	1.26	21.8

**Table 5:** Thermal properties of PBS and PLA–PBS blend formulations plasticized with ISE and ATBC at different contents.

Formulations	*T*_g_ (°C)	*T*_cc_ (°C)	Δ*H*_cc_ (J/g)	*T*_m_ (°C)	Δ*H*_m_ (J/g)	*X*_c_ (%)	*T*_c_ (°C)
1st Heating Scan							Cooling
PBS	41.9 ± 0.2	87.4 ± 0.4	13.1 ± 2.0	114.3 ± 0.1	79.9 ± 0.6	60.6 ± 2.4	79.1 ± 0.6
PBS85–ATBC15	41.9 ± 1.4	87.2 ± 0.2	13.8 ± 2.1	113.0 ± 0.4	77.9 ± 0.6	68.3 ± 2.8	75.1 ± 0.4
PBS80–ATBC20	41.5 ± 8.8	90.1 ± 0.5	11.9 ± 0.7	112.8 ± 0.2	70.9 ± 2.7	66.8 ± 2.3	73.0 ± 0.1
PBS85–ISE15	41.1 ± 0.6	88.1 ± 0.9	12.5 ± 2.1	113.2 ± 0.1	75.8 ± 0.2	67.3 ± 2.0	75.8 ± 0.6
PBS80–ISE20	41.5 ± 0.3	90.6 ± 0.2	12.9 ± 1.4	111.8 ± 0.1	69.7 ± 0.5	64.4 ± 2.2	74.6 ± 0.1
PBS80–ISE30	42.5 ± 0.2	90.5 ± 0.2	11.0 ± 0.3	111.8 ± 0.6	66.2 ± 1.7	71.6 ± 2.6	74.1 ± 0.5
	***T*_g PLA_ (°C)**	***T*_m PBS_ (°C)**	***T*_cc_ (°C)**	***T*_m1_ (°C)**	***T*_m2_ (°C)**		
PLA80–PBS20	56.6 ± 0.5	113.0 ± 0.4	106.6 ± 0.4	147.0 ± 0.3	153.0 ± 0.3		
(PLA15–ISE15)–PBS20	34.3 ± 0.2	110.6 ± 0.1	79.1 ± 0.1	134.6 ± 0.2	147.3 ± 0.3		
(PLA80–PBS20)–ISE15	32.8 ± 0.4	111.0 ± 0.3	78.4 ± 0.4	134.5 ± 0.4	146.8 ± 0.5		
2nd Heating Scan							
**Formulations**	**T_cc_ (°C)**	**ΔH_cc_ (J/g)**	**T_m_ (°C)**	**ΔH_m_ (J/g)**	**X_c_ (%)**		
PBS	102.3 ± 0.1	8.4 ± 0.8	113.7 ± 0.3	69.5 ± 0.9	55.5 ± 0.1		
PBS85–ATBC15	97.3 ± 0.3	9.4 ± 1.8	112.5 ± 0.1	70.5 ± 4.2	65.2 ± 6.5		
PBS80–ATBC20	95.6 ± 0.4	9.4 ± 0.5	111.8 ± 0.2	65.9 ± 5.2	64.8 ± 5.3		
PBS85–ISE15	98.5 ± 0.4	7.3 ± 0.1	112.7 ± 0.1	71.2 ± 2.7	68.1 ± 2.8		
PBS80–ISE20	96.4 ± 0.2	8.7 ± 0.5	111.3 ± 0.1	66.6 ± 2.1	65.5 ± 3.0		
PBS80–ISE30	96.5 ± 0.5	9.0 ± 1.0	111.2 ± 0.6	61.1 ± 3.1	67.5 ± 5.5		
	***T*_g PLA_ (°C)**	***T*_m PBS_ (°C)**	***T*_cc_ (°C)**	***T*_m1_ (°C)**	***T*_m2_ (°C)**		
PLA80–PBS20	55.3 ± 0.4	113.1 ± 0.2	109.3 ± 0.3	147.4 ± 0.4	152.9 ± 0.5		
(PLA15–ISE15)–PBS20	34.7 ± 0.3	111.0 ± 0.1	87.1 ± 0.1	135.5 ± 0.2	147.1 ± 0.4		
(PLA80–PBS20)–ISE15	34.7 ± 0.3	111.3 ± 0.1	87.6 ± 0.4	135.5 ± 0.4	147.1 ± 0.4		

**Table 6:** Mechanical properties of plasticized PLA, PBS and PLA–PBS blend based formulations.

Formulations	*E*_Young_ (MPa)	σ_Y_ (MPa)	ε_Y_ (%)	σ_B_ (MPa)	ε_B_ (%)
PLA based plasticized films					
PLA	2777 ± 242	46.4 ± 7.3	1.9 ± 0.3	43.6 ± 7.3	5.4 ± 2.1
PLA85–ISE15	768 ± 166	10.3 ± 2.6	2.8 ± 0.2	22.3 ± 5.8	208.6 ± 58.4
PLA80–ISE20	312 ± 3	5.4 ± 0.2	2.9 ± 1.7	15.7 ± 2.3	196.1 ± 35.3
PLA70–ISE30	315 ± 15	5.7 ± 1.1	2.5 ± 0.4	11.7 ± 3.2	160.3 ± 85.7
PLA85–ATBC15	272 ± 69	4.1 ± 0.4	3.3 ± 0.3	14.1 ± 4.7	228.6 ± 59.9
PLA80–ATBC20	167 ± 87	2.4 ± 1.3	3.2 ± 0.5	9.8 ± 3.1	199.1 ± 5.6
PLA70–ATBC30	123 ± 10	5.1 ± 0.3	12.4 ± 1.1	13.5 ± 1.1	181.9 ± 19.9
PBS based plasticized films					
PBS	501 ± 108	-	-	24.2 ± 2.2	9.7 ± 1.9
PBS85–ISE15	350 ± 18	16.7 ± 0.6	8.4 ± 0.4	20.9 ± 0.4	29.6 ± 3.2
PBS80–ISE20	311 ± 23	15.2 ± 0.8	8.3 ± 0.9	18.1 ± 1.1	26.2 ± 7.6
PBS70–ISE30	276 ± 6	13.7 ± 0.4	8.2 ± 0.4	16.6 ± 0.8	28.6 ± 5.9
PBS85–ATBC15	310 ± 68	11.2 ± 1.1	4.6 ± 0.3	18.5 ± 1.5	14.6 ± 1.2
PBS80–ATBC20	275 ± 25	-	-	6.3 ± 1.1	3.7 ± 2.5
PLA–PBS plasticized blend films					
PLA80–PBS20	2405 ± 109	43.5 ± 2.2	2.1 ± 0.1	33.7 ± 2.8	10.5 ± 5.4
(PLA85–ISE15)–PBS20	758 ± 53	11.5 ± 0.4	4.6 ± 1.9	19.9 ± 3.2	241.6 ± 37.6
(PLA80–PBS20)–ISE15	511 ± 121	11.1 ± 1.3	4.8 ± 1.7	19.8 ± 2.5	249.5 ± 19.4

**Table 7:** Contact angle (CA) values of plasticized PLA–PBS based formulations.

Materials	CA (°)
PLA based plasticized films	
PLA	68 ± 2
PLA85–ISE15	65 ± 1
PLA–PBS plasticized blend films	
PLA80–PBS20	61 ± 1
(PLA85–ISE15)–PBS20	56 ± 1
(PLA80–PBS20)–ISE15	56 ± 2
